# Suspended Liquid Subtractive Lithography: One-step generation of 3D channel geometries in viscous curable polymer matrices

**DOI:** 10.1038/s41598-017-07630-w

**Published:** 2017-08-07

**Authors:** D. Helmer, A. Voigt, S. Wagner, N. Keller, K. Sachsenheimer, F. Kotz, T. M. Nargang, B. E. Rapp

**Affiliations:** Karlsruhe Institute of Technology, Institute of Microstructure Technology IMT, Hermann-von-Helmholtz-Platz 1, 76344 Eggenstein-Leopoldshafen, Germany

## Abstract

The miniaturization of synthesis, analysis and screening experiments is an important step towards more environmentally friendly chemistry, statistically significant biology and fast and cost-effective medicinal assays. The facile generation of arbitrary 3D channel structures in polymers is pivotal to these techniques. Here we present a method for printing microchannels directly into viscous curable polymer matrices by injecting a surfactant into the uncured material via a steel capillary attached to a 3D printer. We demonstrate this technique using polydimethylsiloxane (PDMS) one of the most widely used polymers for the fabrication of, e. g. microfluidic chips. We show that this technique which we term Suspended Liquid Subtractive Lithography (SLSL) is well suited for printing actuators, T-junctions and complex three dimensional structures. The formation of truly arbitrary channels in 3D could revolutionize the fabrication of miniaturized chips and will find broad application in biology, chemistry and medicine.

## Introduction

Today, most polymer systems for life science applications are manufactured using replication techniques, where a structure is created as the inverse of a master or mould. Polydimethylsiloxane (PDMS) remains the material of choice for chip manufacturing in life science applications since it is non-toxic, biocompatible, transparent and elastomeric^1^. These chips commonly incorporate passive microfluidic channels as well as membrane valve actuators for fluid control. Miniaturization is a promising strategy for resource-saving and sustainable chemistry and life-sciences but it has not yet reached its full potential. This is largely due to slow production processes and limited channel design possibilities. The need for master formation to generate chip structures significantly increases processing time and costs and severely restricts channel geometries: Three dimensional channels can only be approximated by stacking of individual layers^2^ or lost-form-molding of three dimensional scaffolds^3–5^. Replicated structures are sealed by bonding a lid onto the channel structure, a process that is difficult to control, limits the pressure resistance and restricts the geometry to a two-dimensional landscape and channel cross-sections with a flat top^6, 7^. New methods which allow the formation of channels with circular cross-sections which are essential for mimicking biological environments such as blood vessels or capillaries have been sought for a long time^8–13^. As of today there is no method which allows for direct 3D printing of channels. Fugitive Inks may be printed by a 3D printer but are restricted to multilayer two dimensional structures and require consecutive embedding into polymer matrices^14, 15^. Embedded printing of conductive inks into standard elastomers is also restricted to two dimensional multilayers^16^. Printing of truly three-dimensional structures has so far only been demonstrated by printing a polymeric ink into granular gels^17^ a process which is not compatible with standard polymer matrices and thus does not lend itself to chip manufacturing.

In this paper, we demonstrate a novel process we term Suspended Liquid Subtractive Lithography (SLSL) that allows for direct writing of three dimensional, circular channels into viscous curable polymer matrices. We demonstrate this process using PDMS as polymer of choice and a RepRap 3D printing system for dispensing a commercially available non-toxic surfactant directly into the curing polymer matrix thus generating freely definable three-dimensional channel structures. Uniform channels between 200 µm and 500 µm in diameter have been produced with a 150 µm capillary without the need of a replication master, photolithography, or device sealing and bonding. SLSL is a simple one-step manufacturing process which allows for the creation of truly free-form 3D channel layouts in polymers within a few minutes of printing time.

## Results

**Figure 1:**
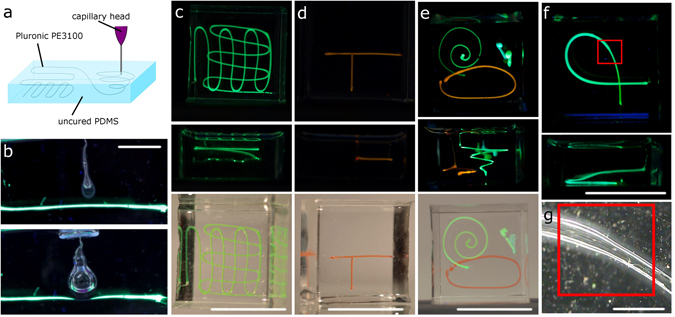
3D printing of microfluidic channels in PMDS using Suspended Liquid Subtractive Lithography (SLSL). (**a**) Schematic of the SLSL method for printing channels directly into PDMS by injecting Pluronic PE3100 surfactant into uncured Elastosil 601. Printed channels were cleared from Pluronic PE3100 and filled with an aqueous solution of dye (green and orange). (**b**) Quake- type membrane actuator, with a fluid channel (bottom, green) and an expandable control channel on top. When the control channel is pressurized the channel expands, thus blocking the fluid channel, scale bar: 5 mm. Top images of (**c**,**d**,**e**,**f**) show the top view of the structure illuminated at 365 nm, middle images show the side view illuminated at 356 nm and bottom images show the structures at ambient light. (**c**) meander-like channel with two interconnected layers, scale bar: 18 mm, (**d**) T-junction, scale bar: 18 mm, (**e**) arbitrary structure of several z-layers and a three dimensional spiral, scale bar: 18 mm, (**f**) z-loop, scale bar: 18 mm, (**g**) close up of channel shown in (**f**) with a smooth transition between channel diameter of 550 µm and 350 µm manufactured by increasing the amount of extruded surfactant, scale bar: 1 mm.

To achieve channel formation directly inside the curing viscous PDMS matrix the liquid deposited by SLSL (see Fig. 1a) has to meet the following criteria: (1) a density close to the density of cured PDMS to prevent sedimentation or floating, (2) low surface energy to prevent formation of droplets, (3) low viscosity to simplify extrusion through a capillary and (4) biocompatibility as well as low toxicity to allow for printing in a standard laboratory. Pluronic PE3100, a surfactant with a density of 1.02 g/mL and a viscosity of 175 cP meets all aforementioned criteria and was used to print actuators (Fig. 1b), meander-like structures (Fig. 1c), T-junctions (Fig. 1d) and three dimensional structures like spirals (Fig. 1e) and loops (Fig. 1f) into Elastosil 601. Pluronic PE3100 does not form droplets inside the PDMS matrix due to the close match in surface tension between the two liquids. Droplets will develop due to Rayleigh-Plateau instability only if there is a mismatch in surface tension which gives rise to Young-Laplace forces curving the interface.

A customized RepRap Pro printing system was used for printing (see Supplementary Fig. S1). The printer was equipped with a 150 µm capillary and an external extrusion system. The capillary was attached to the printing unit and connected via a low-elasticity tube to a custom-made syringe pump. The plane of the table is referred to as x/y-plane, the z-direction points upwards. The following definitions are made to clarify the process parameters of the RepRap printer (see Fig. S1a): the feedrate F is the “writing speed”, the speed at which the print head (see Fig. S1b) and thus the needle moves through the PDMS. The distances between coordinates in the x/y/z space is defined as distance d. The extrusion E is defined as the steps of the extrusion stepper motor (see Fig. S1c) that lead to an extrusion of 1 mm filament (in the usual printing process with polymer filament). The extrusion factor e is defined as the length of filament [mm] extruded per mm of head movement, thus e = E/d. The extrusion factor e usually remains constant throughout the writing process. A 1.8° stepper motor (thus 200 steps per full 360° rotation of the spindle) with 16x software-induced microstepping (16 * 200 steps = 3200 steps per 360° movement of the threaded bar) was used. Channel diameters can be controlled by adjusting the amount of extruded surfactant, i. e. by changing the extrusion factor e. Since the RepRap system is designed for printing polymer filaments, the extrusion E (mm filament going into the extrusion nozzle) is an arbitrary parameter when it comes to extruding liquids. The motor control driver was set to 16-fold microstepping (3200 steps per 360°). The motor setting was set such that 20 steps of the extrusion motor (e-steps) lead to an E value of 1.000 thus a theoretical amount of 1 mm filament moving into the extruder. The e factor, the extrusion E per distance d covered by the writing head, i. e. capillary, e = E/d was set to 0.2. These setting ensure a very low amount of steps of the motor for the extrusion of the liquid: 20 * 0.2 = 4 steps of the motor (corresponding to a movement of 0.45° of the motors threaded bar) for a theoretical extrusion of 1 mm of filament. These settings resulted in approximately 0.2 µL of surfactant extruded per mm traversed by the extrusion head/capillary.

Important parameters influencing the printing results are viscosity and curing time of the PDMS matrix, writing speed of the moving capillary, amount of extruded surfactant and capillary length. These factors will be discussed in the following paragraphs.

**Figure 2:**
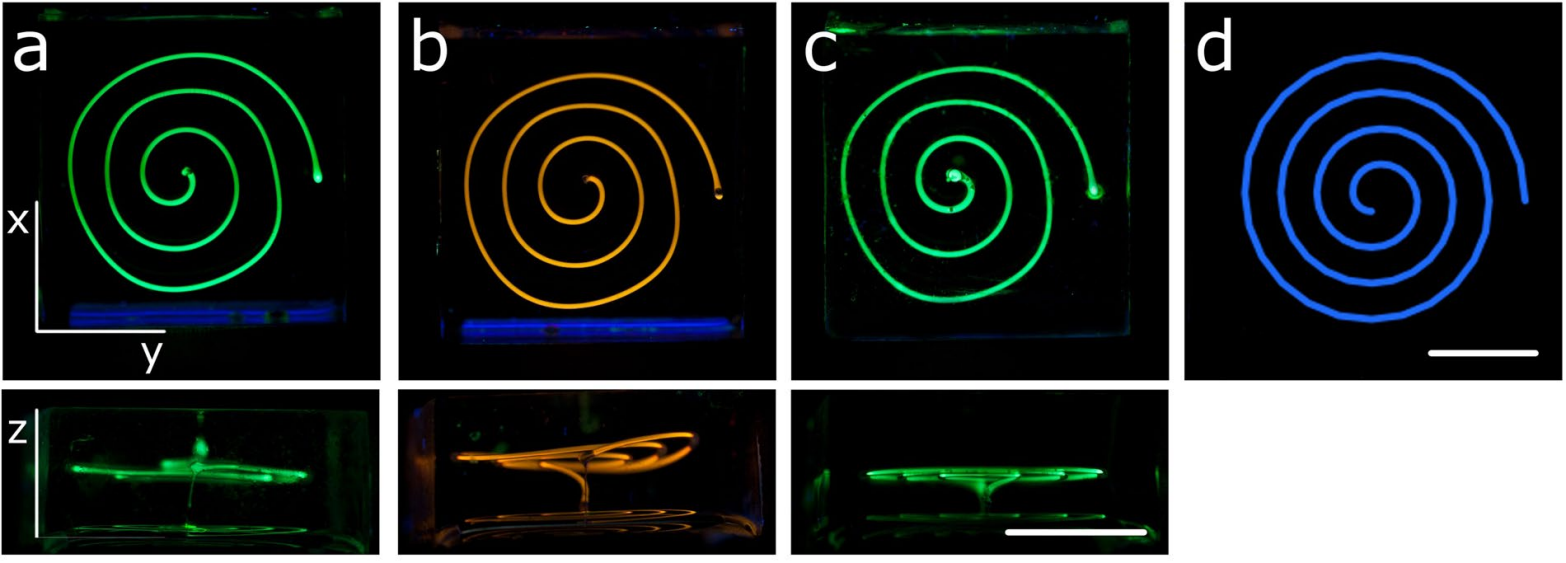
Distortions of printed structures in the z-plane and x/y-plane. Spirals of 400 µm diameter were printed at 50 mm/min writing speed at (**a**) 6 min, (**b**) 15 min, and (**c**) 30 min PDMS curing time. The distortions in the z-plane occur due to sedimentation effects, higher curing time leads to more stability of the z-layers. Distortions in the x/y-plane occur due to the high viscosity of the PDMS matrix: (**d**) shows the original G-file image of structures (**a**–**c**). Channels were filled with an aqueous solution of dye and illuminated at 365 nm to increase visibility. Scale bars: 10 mm.

The viscosity of this PDMS mixture increases at a rate of 53 ± 3 cP/min during the first 30 min of room temperature curing (see Supplementary Fig. S2). Thus, the printing has to be sufficiently quick to ensure similar conditions for all printed channels. Distortions of the printed structures may occur in z- as well as in x/y-direction. In z-direction distortions are caused by density effects, i. e. sedimentation of the Pluronic PE3100 surfactant during the curing process. PDMS shrinks upon curing and thus its density increases to a final value of 1.02 g/mL. Since the surfactant also possesses a density of 1.02 g/mL it is denser than the matrix when first printed. Therefore a suitable curing time has to be chosen that ensures minimal sedimentation effects. We found a PDMS curing time of 30 min leads to the highest stability of the z-plane, (see Fig. 2a–c). Exposing the PDMS to elevated temperatures or infrared light leads to an uneven curing process, where the surfaces of the PDMS block cure significantly faster than the bulk. PDMS blocks were therefore cured at room temperature (22–23 °C) overnight to ensure an evenly cured material with planar z-layers. Distortions in x/y directions occur due to viscosity of the PDMS matrix that applies significant force onto the printed liquid. Especially in curved structures, the forces lead to a distortion of the overall channel length and a reduced contour accuracy (see Fig. 1d). The liquid-in-liquid printing technique however ensures extremely smooth channel walls as confirmed by scanning electron microscopy (see Fig. S3). Further complex di-helical structures printed by SLSL are displayed in Fig. S4.

**Figure 3:**
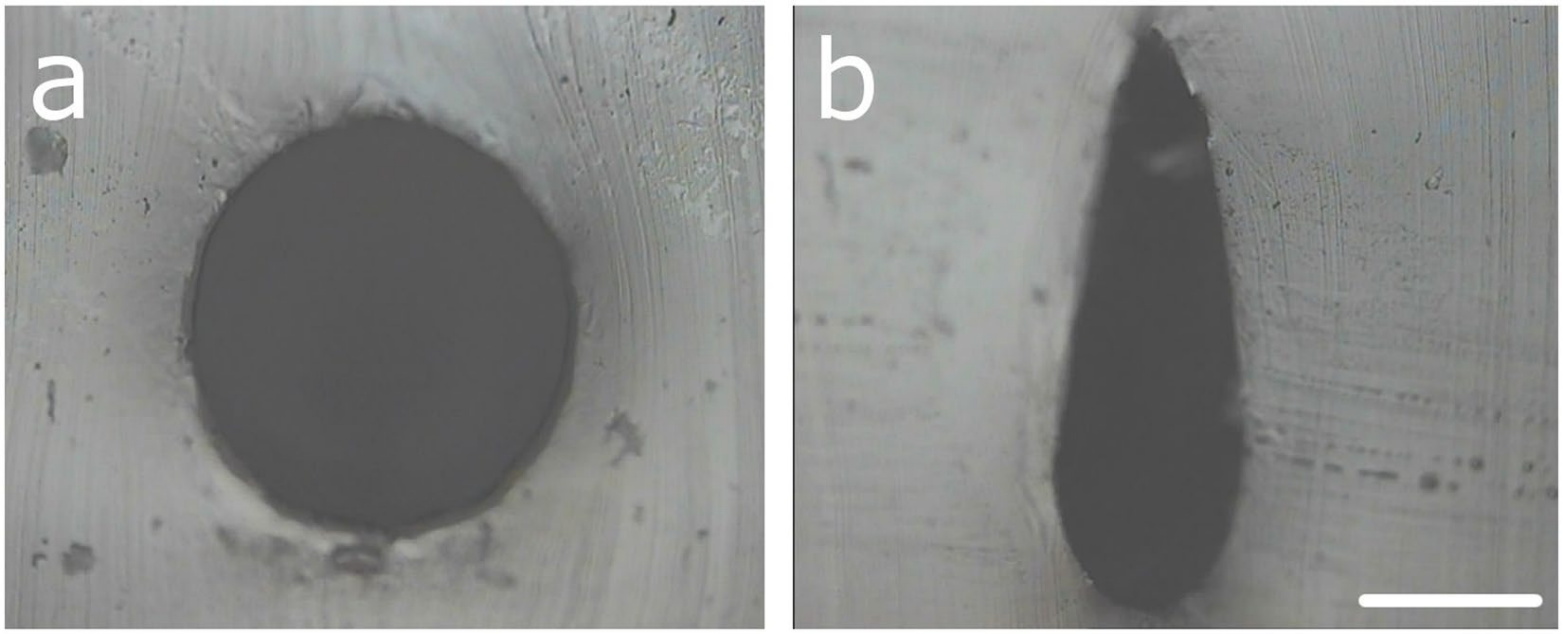
Variation of the channel cross-section and high-aspect ratio channels by capillary wetting effects. (**a**) Circular cross-section achieved by nearly simultaneous start of extrusion and capillary movement. (**b**) High-aspect ratio channels achieved by a delay in capillary movement compared to the start of the extrusion. Scale bar: 250 µm.

The shape of the printed channels can be influenced by exploiting the wetting properties of the capillary. When the capillary is immobile at the first extrusion of surfactant, the surfactant wets the needle, leading to an oval or drop-shaped distortion of the otherwise circular channels (see Fig. 3). This effect can be used to create microfluidic channels with high aspect ratios in PDMS which is important for e.g. distributive fluidic mixing applications. Figure 3b shows a drop-shaped channel cross-section with an aspect ratio of 3:1.

**Figure 4:**
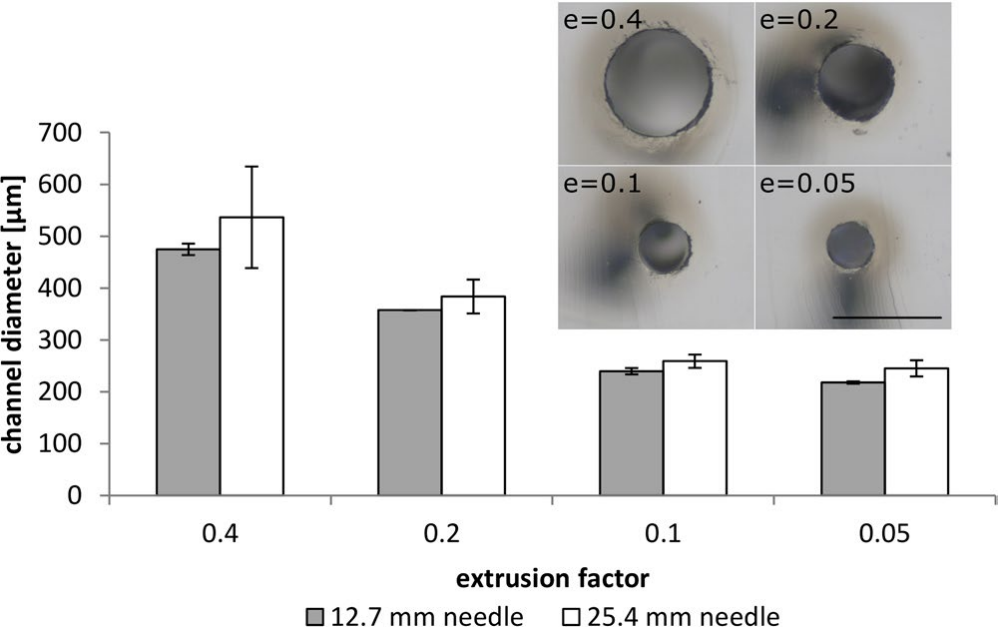
Influence of the extrusion factor and needle length on channel diameters and diameter variations. Diameter of channels printed at 50 mm/min with different extrusion factor settings and two different needle lengths (internal diameter of both needle types: 0.15 mm). Standard deviations of channel diameters are much higher when using a longer 25.4 mm (1 inch) needle. Inset: Channel diameters of channels printed at the different extrusion factor settings with the 12.7 mm (0.5 inch) needle, scale bar 500 µm.

Channel diameters can be varied by adjusting the amount of extruded surfactant, i. e. by changing the extrusion factor *e* (see Supplementary Information for details on the printing parameters of the RepRap instrument). The printing depth is limited by the needle length and investigated in the following. Four straight lines were printed into the same PDMS mixture with different extrusion factors: 0.4, 0.2, 0.1 and 0.05. With these extrusion factors, channel diameters of 475 µm, 358 µm, 240 µm, 218 µm were measured for a capillary of 12.7 mm (0.5 inch) length, and 537 µm, 384 µm, 259 µm, 245 µm for a capillary of 25.4 mm (1 inch) length. Thus the minimum channel diameter for printing with a 150 µm capillary is approximately 220 µm. Printing with a shorter capillary yields much more uniform channel diameters. This is most likely due to greater distortion effects on the longer needle caused by the high viscosity of the matrix. In a single print, channel diameter variations between 12.7 mm and 25.4 mm needle were comparable (see Fig. 4). Extrusion factors can also be changed in a single printing experiment, leading to a smooth transition in channel thickness (see Fig. 1g). Data on the thickness of individual channels and the variation in mean channel diameters are shown in Figs S5 and S6.

After curing the surfactant is removed from the channel network by purging with solvent. To ensure that Pluronic PE3100 was effectively removed fro﻿m the channels, mass spectrometric analysis of solvents used for washing was performed. The detection limit of PE3100 traces was determined to be at a concentration of 170 nM (see Supplementary Fig. S7). A channel of 18 mm length and 300 µm width was determined to be surfactant-free according to the detection limits after pumping of 5 mL 2-propanol through the channel (see Supplementary Fig. S8).

In conclusion we have developed a novel method termed Suspended Liquid Subtractive Lithography (SLSL) which allows rapid prototyping of smooth, truly three-dimensional channel geometries directly into viscous curable polymer matrices using a commercially available, low-cost 3D printing system. Using PDMS as an exemplary polymer we obtained the best results when printing Pluronic PE3100 into the polymer matrix after 30 min curing time. Writing speed of 50 mm/min was found appropriate to minimize shear stresses upon printing. Different channel diameters between 200 µm and 500 µm could be printed by varying the extrusion speed of the surfactant. SLSL is a simple and rapid one-step alternative to complex and time-consuming multi-step replication processes and offers significantly increased freedom of design. SLSL is the first method compatible with mass-production of polymer chips and will allow device miniaturization to reach its full potential.

## Methods

Wacker Elastosil 601, a clear, room-temperature curing PDMS was used for all experiments. A RepRap Pro Ormerod 2 3D printing system with an attached custom-made syringe pump was used for printing. Pluronic PE3100 surfactant was purchased from the BASF (Germany). 0.15 mm internal diameter needles (25.4 mm and 12.7 mm in length) for the extrusion system were purchased from Vieweg (Germany). The dye used for filling the channels for visualization purposes was extracted from text markers (Faber Castell Textliner 1548) refilling cartouches by immersion in water.

### Customization of RepRap Pro

The stepper motor for polymer extrusion was used to operate a glass syringe pump, connected with a PTFE tube to the x-plane arm motor sledge which was equipped with a syringe and a needle. The motor control driver was set to 16-fold microstepping (3200 steps per 360°). Luer-Lock was used for all fluidic connections to prevent leakages.

### Determination of extrusion rate

The mass of three microcentrifuge tubes was determined individually. The RepRap instrument was set to a feedrate *F* of 50 mm/min and an extrusion *E* of 1 mm. The extrusion motor was set to 20 steps for the extrusion of 1 mm filament and the extrusion factor *e* = *E/d* was set to 0.2. When the surfactant started to drip from the needle, the needle was cleaned, the timer was started and the microcentrifuge tube was placed under the needle. Surfactant was collected in the tube for 4 to 4.5 min. The mass of the tubes was determined and the extrusion rate was calculated.

### Preparation of PDMS

Wacker Elastosil 601 PDMS was used for all experiments. It comprises component A, containing the platinum catalyst, with a viscosity of 5000 cP and a density of 1.03 g/mL and component B, containing the crosslinker, with a viscosity of 40 cP and a density of 0.97 g/mL. Upon mixing in a 9:1 weight ratio (A:B), the viscosity of the mix is 3500 cP. The cured PDMS possesses a density of 1.02 g/mL. Around 7.6 g of Elastosil 601 component A was weighed into a 100 mL beaker. Component B [m(B) = m(A)/9] was pipetted onto component A immediately after the timer was started. The two components were stirred vigorously for 20 s by hand and consecutively placed in a small (18 cm) desiccator connected to a vacuum pump. The mixture was degassed by vacuuming and venting the mix until the timer had reached 4 min. The mix was directly poured into 2.4 cm * 2.4 cm * 1.6 cm (l * w * h) wells that were filled roughly to 0.9 cm height.

### Printing in PDMS

The PDMS filled well was placed on the x/y-table of the RepRap Pro Ormerod 2 instrument and secured by glued-on holders. The extrusion speed was regulated by the extrusion factor *e*. The setting used for regular printing was 0.2. Roughly 1 min before starting the print the needle was placed into the PDMS.

### Viscosity measurements

Viscosity of Elastosil 601 upon curing was measured using a Brookfield LVDV-II Pro with a CPE-51 spindle at 1 rpm (23 °C). PDMS was prepared as described above, and roughly 500 µL of the mixture was poured into the instrument container. To prevent heating during measurements, the container was opened between measurements.

### Microscopy

Channels were cut open with a scalpel and placed under a reflected-light microscope Zeiss Axiotec 100 HD with a Panasonic wv_GP230 camera or a Zeiss Stemi 508 stereomicroscope.

## Electronic supplementary material

Supplementary Information
