# Supplementary material for: Suspended Liquid Subtractive Lithography: One-step generation of 3D channel geometries in viscous curable polymer matrices
Source: Sci Rep. 2017 Aug 7;7:7387. doi: 10.1038/s41598-017-07630-w (PMC5547044; doi:10.1038/s41598-017-07630-w)
Supplement: Supplementary file 1 — Supplementary Information [file 41598_2017_7630_MOESM1_ESM.doc]

**Supplementary Information**

for

**Suspended Liquid Subtractive Lithography: One-step generation of 3D channel geometries in viscous curable polymer matrices**

D. Helmer^a^, A. Voigt^a^, S. Wagner^a^, N. Keller^a^, K. Sachsenheimer^a^, F. Kotz^a^, T. M. Nargang^a^, B. E. Rapp^a^*

a Karlsruhe Institute of Technology KIT, Institute of Microstructure Technology IMT, Hermann-von-Helmholtz-Platz 1, 76344 Eggenstein-Leopoldshafen.

*bastian.rapp@kit.edu


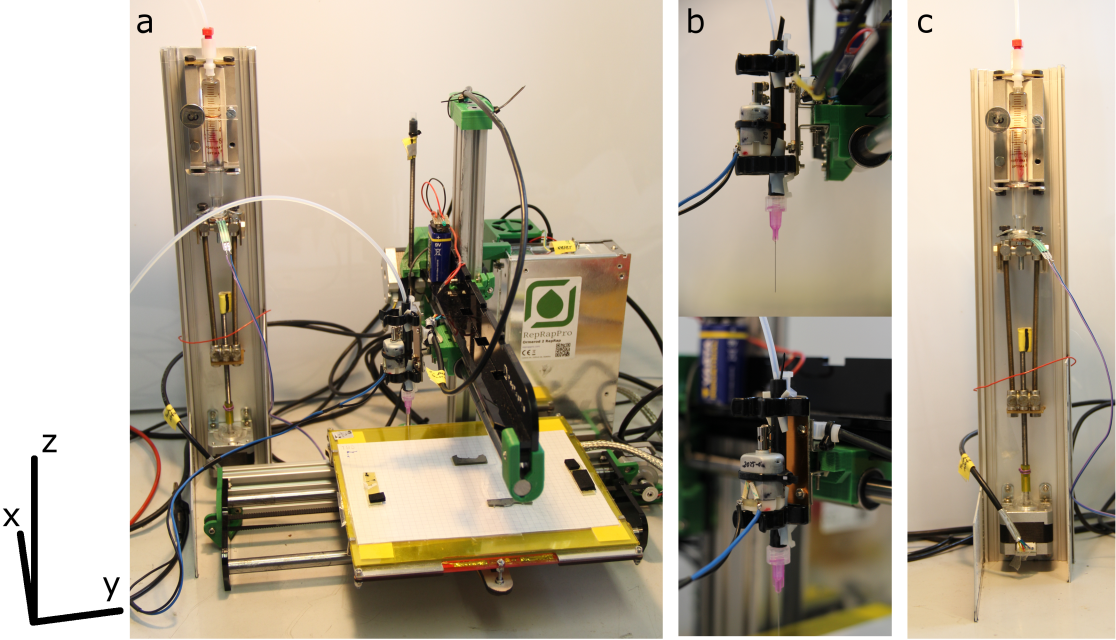


Figure S1: Customized RepRapPro Ormerod 2 3D printer used for printing. a) Full view of the printing unit, syringe pump and capillary holder. b) Close-up of the customized printing head equipped with a capillary holder. c) Custom-made syringe pump. The plane of the table corresponds to x/y, movements upwards are in z-direction.


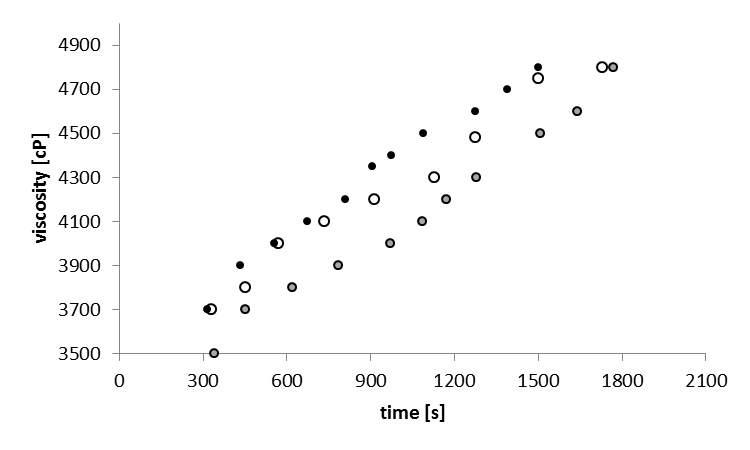


Figure S2: Increase of viscosity (Δη) of a A:B=9:1 mixture of PDMS (7.6 g component A and 0,85 g component B) during the first 30 min of curing. An increase of roughly 53±3 cP per minute was determined from the slope of the linear regression fits.


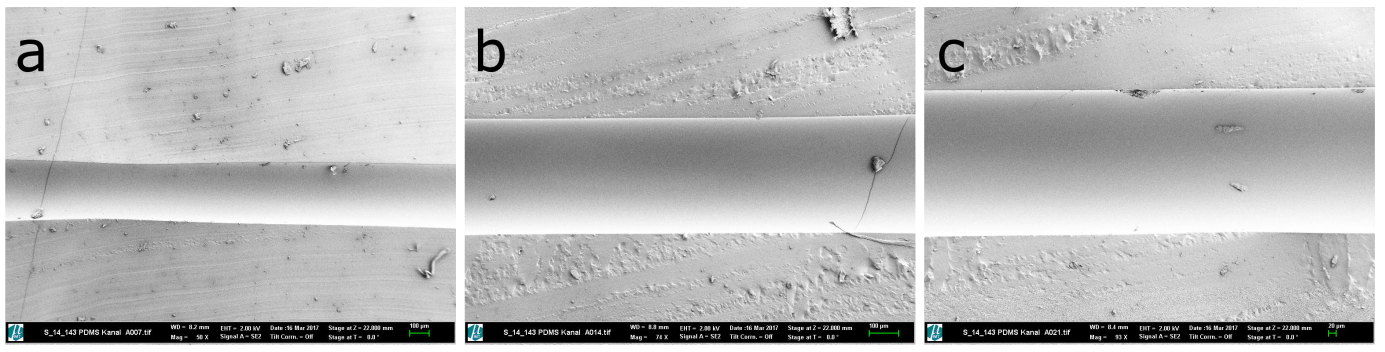


Figure S3 SEM images of a) 300 µm, b) 390 µm and c) 180 µm channels printed by SLSL. Due to the interface built by two immiscible liquids, the walls of the channels are extremely smooth.


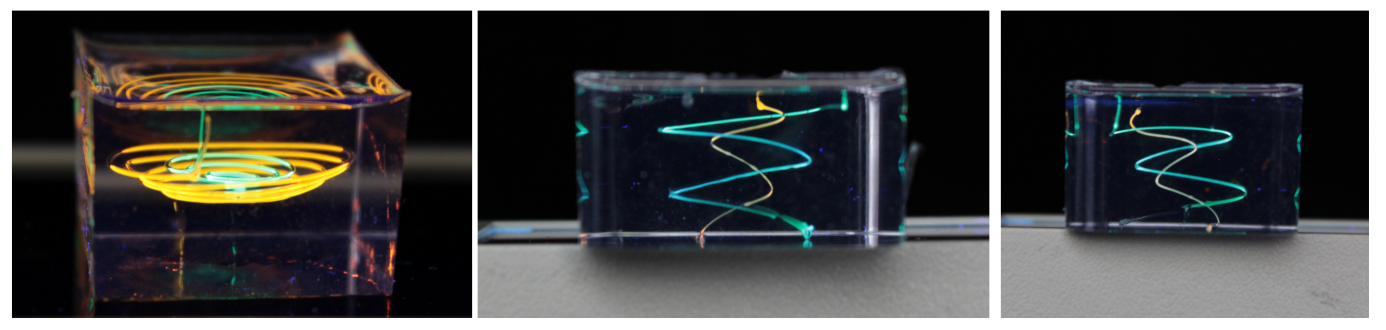


Figure S4: Complex three dimensional double helical SLSL structures. SLSL is suitable also for intricate microfluidic structures.


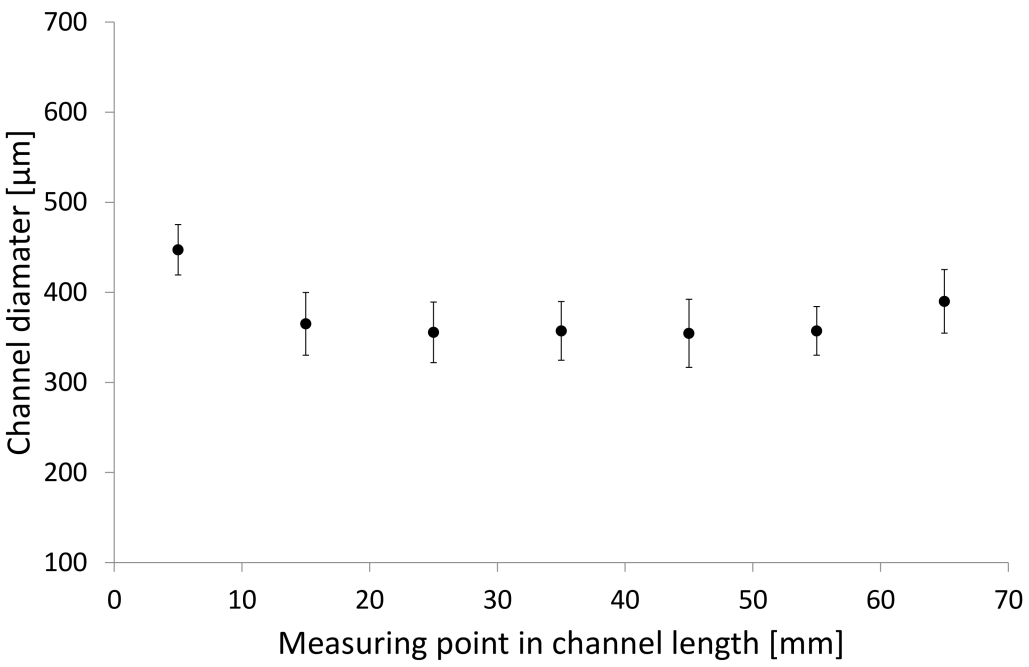


Figure S5: Channel diameter over the length of nine 60-65 mm channels. At the start and at the end of the writing head movement, the acceleration increases and decreases. The extruder does not effectively correct for this, therefore the channel thickness increases towards the channel endpoints.


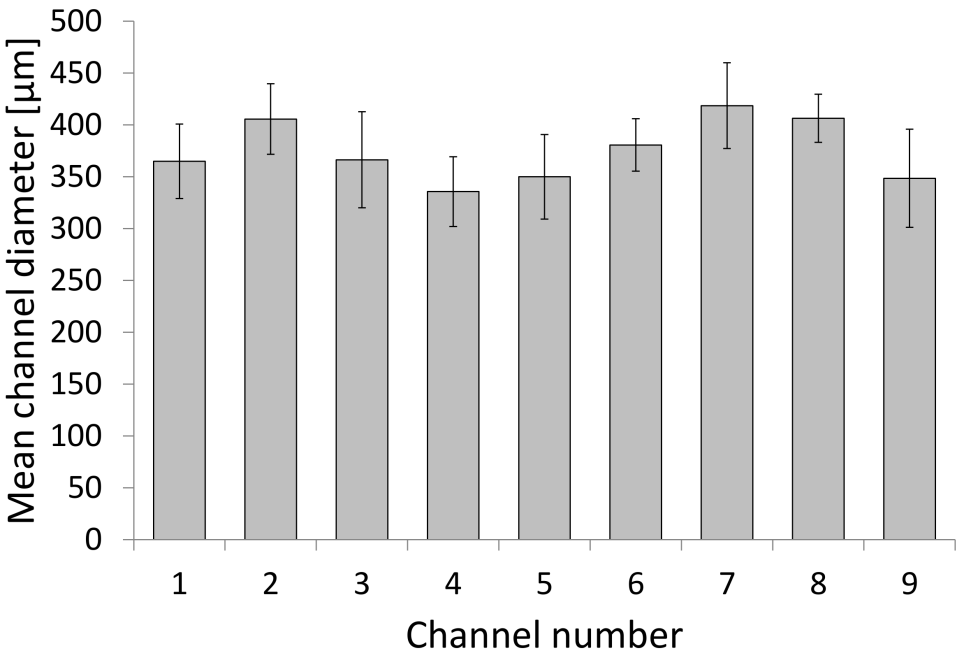


Figure S6: Mean channel diameter and standard deviation from channels 1-9 shown in Figure S5. Channel thicknesses vary between 10 µm and 50 µm over a channel length of 60-65 mm.


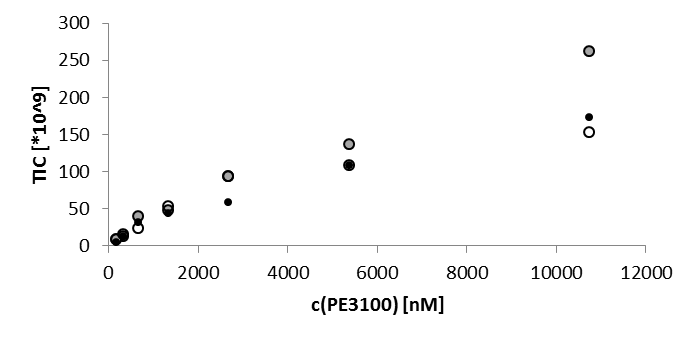


Figure S7: Calibration curves for detection limit of traces of Pluronic PE3100 measured by APCI (+) mass spectrometry. The detection limit was determined to be 170 nM Pluronic PE3100 in methanol corresponding to a total ion count (TIC) of 7±3 *10^9^.


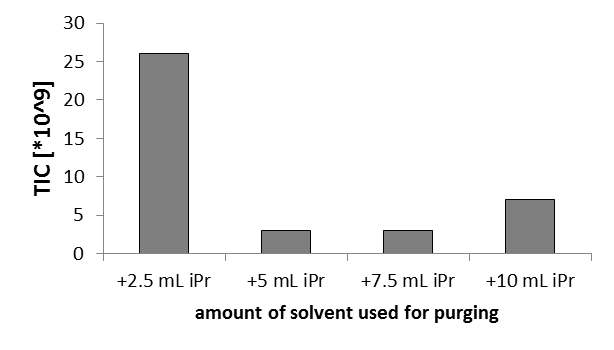


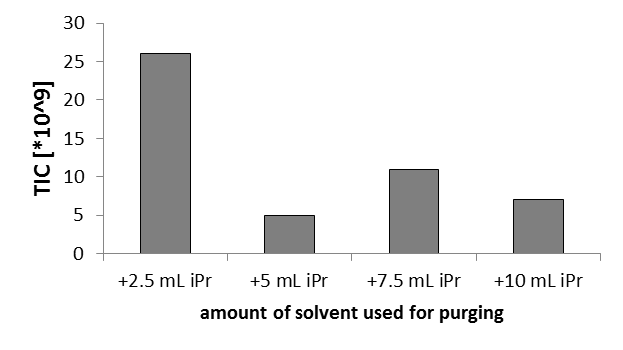


Figure S8: Mass spectrometric analysis of residual PE3100 after purging a 1.8 cm straight channel of 500 µm diameter with different amounts of 2-propanol. After purging with > 5 mL of 2-propanole the amount of residual PE3100 remains constant within the detection limit (total ion count of 7±3 *10^9^).
